# An increase in the burden of neonatal admissions to a rural district hospital in Kenya over 19 years

**DOI:** 10.1186/1471-2458-10-591

**Published:** 2010-10-06

**Authors:** Michael K Mwaniki, Hellen W Gatakaa, Florence N Mturi, Charles R Chesaro, Jane M Chuma, Norbert M Peshu, Linda Mason, Piet Kager, Kevin Marsh, Mike English, James A Berkley, Charles R Newton

**Affiliations:** 1 Centre for Geographic Medicine Research (Coast), Kenya Medical Research Institute, PO Box 230, Kilifi, Kenya; 2 Liverpool University, Liverpool, UK; 3 Department of Infectious Diseases, Tropical Medicine and AIDS, Academic Medical Centre, University of Amsterdam, Amsterdam, the Netherlands; 4 Nuffield Department of Clinical Medicine, University of Oxford, John Radcliffe Hospital, Oxford, UK; 5 Centre for clinical Vaccinology and Tropical medicine, Churchill Hospital, Oxford University, Oxford, UK; 6 Clinical Research Unit, London School of Hygiene and Tropical Medicine, London, UK; 7 Neurosciences Unit, Institute of Child Health, The Wolfson Centre, Mecklenburgh Square, London, WC1N 2AP, UK

## Abstract

**Background:**

Most of the global neonatal deaths occur in developing nations, mostly in rural homes. Many of the newborns who receive formal medical care are treated in rural district hospitals and other peripheral health centres. However there are no published studies demonstrating trends in neonatal admissions and outcome in rural health care facilities in resource poor regions. Such information is critical in planning public health interventions. In this study we therefore aimed at describing the pattern of neonatal admissions to a Kenyan rural district hospital and their outcome over a 19 year period, examining clinical indicators of inpatient neonatal mortality and also trends in utilization of a rural hospital for deliveries.

**Methods:**

Prospectively collected data on neonates is compared to non-neonatal paediatric (≤ 5 years old) admissions and deliveries' in the maternity unit at Kilifi District Hospital from January 1^st ^1990 up to December 31^st ^2008, to document the pattern of neonatal admissions, deliveries and changes in inpatient deaths. Trends were examined using time series models with likelihood ratios utilised to identify indicators of inpatient neonatal death.

**Results:**

The proportion of neonatal admissions of the total paediatric ≤ 5 years admissions significantly increased from 11% in 1990 to 20% by 2008 (trend 0.83 (95% confidence interval 0.45 -1.21). Most of the increase in burden was from neonates born in hospital and very young neonates aged < 7days. Hospital deliveries also increased significantly. Clinical diagnoses of neonatal sepsis, prematurity, neonatal jaundice, neonatal encephalopathy, tetanus and neonatal meningitis accounted for over 75% of the inpatient neonatal admissions. Inpatient case fatality for all ≤ 5 years declined significantly over the 19 years. However, neonatal deaths comprised 33% of all inpatient death among children aged ≤ 5 years in 1990, this increased to 55% by 2008. Tetanus 256/390 (67%), prematurity 554/1,280(43%) and neonatal encephalopathy 253/778(33%) had the highest case fatality. A combination of six indicators: irregular respiration, oxygen saturation of <90%, pallor, neck stiffness, weight < 1.5 kg, and abnormally elevated blood glucose > 7 mmol/l predicted inpatient neonatal death with a sensitivity of 81% and a specificity of 68%.

**Conclusions:**

There is clear evidence of increasing burden in neonatal admissions at a rural district hospital in contrast to reducing numbers of non-neonatal paediatrics' admissions aged ≤ 5years. Though the inpatient case fatality for all admissions aged ≤ 5 years declined significantly, neonates now comprise close to 60% of all inpatient deaths. Simple indicators may identify neonates at risk of death.

## Background

Each year close to four million newborns die world wide [1,2]. Over 98% of these deaths occur in developing nations with the highest rates in Africa [3]. Many more newborns who survive have brain insults, resulting in severe disabilities such as convulsive disorders, cerebral palsy and cognitive impairments, thus adding further burden to healthcare, social systems and the home environment [4-7].

Over the last two decades sustained resources and effort have been put into prevention and reduction of morbidity and mortality of children aged ≤ 5years. Some of the key areas that have received widespread attention include increasing and sustaining immunisation coverage, introduction of new vaccines for invasive bacterial diseases (haemophilus and pneumococcal infections) and strategies aimed at prevention and development of effective treatment for falciparum malaria [8-11]. These interventions have largely benefited the older child (2 to 60 months) and it is thought mortality in this group may have halved in some areas [1], particularly with the reduction of certain diseases such as malaria and pneumonia [10-13].

Contrary to the encouraging trend in the older child, neonatal morbidity and mortality remains unacceptably high [1-3], with nearly 40% of the annual global deaths in children aged ≤ 5years occurring during the neonatal period [14]. This presents a major obstacle in achieving the aspirations of the fourth millennium development goal of reducing under five mortality by two thirds by the year 2015 in most of the resource poor nations [15]. Paradoxically, despite the fact that these countries have the highest rates of neonatal deaths, there are no published studies demonstrating trends in neonatal admissions and outcome in rural health care facilities in resource poor regions. This issue was highlighted by the World Health Organization (WHO) concerns that neonatal morbidity and mortality rates have largely not been measured long enough to reach reliable conclusions on trends [14]. Such information is critical in planning public health interventions. Importantly, the ability to recognize neonates with potentially life-threatening illness warranting urgent medical attention at presentations to rural health facilities is thought to be critical in reducing mortality [3]. However, current guidelines to recognise such neonates are largely based on data from the WHO young infant study of the early 1990 s [16-18]. A major limitation of this study is that it did not include the first week of life, where most of the neonatal deaths occur.

Therefore, given the paucity of information outlined, we describe trend in neonatal admissions and outcome to a district general hospital in a Kenyan rural area over a 19-year period, and further examine indicators of inpatient neonatal deaths.

## Methods

### Site

This study was done at Kilifi District Hospital (KDH) on the coast of Kenya. The hospital is located in a malaria endemic area and serves a population of over 500,000 people [19], but 80% of the admissions come from an area with 260,000 inhabitants. The hospital has a 40-bed general all purpose paediatric ward with designated areas for nursing various disorders such us acute conditions, paediatric surgery, burns, diarrhoeal diseases, malnutrition and neonatal conditions. In addition the hospital has a 40-bed maternity facility. Newborns delivered with complications or who fall ill post delivery are transferred to the neonatal bay. Likewise neonates born at home and brought to the hospital if ill are admitted in the neonatal bay if thought to be severely unwell. A high dependency unit (6 beds) exists where very severely sick children are managed. Available interventions in the general paediatric ward and the high dependency unit include oxygen, intravenous fluids, antibiotics, phototherapy, exchange transfusion, and nasogastric tube feeding, but not parenteral nutrition, mechanical ventilation or umbilical arterial catheterization. Though the hospital is a district level health facility, very few cases are referred (mainly complex surgical cases) to the provincial hospital. Consent for use of the data was obtained from the guardian of every individual child at point of admission, and the study was approved by Kenyan National Scientific and Ethical Review Board.

### Data collection

#### Pediatric data

At KDH, a prospective surveillance system of all paediatric admissions has been in place since 1989. Based on this system, several studies on malaria, lower respiratory tract infections, malnutrition and neonatal conditions, amongst others, have been published [20-22]. On admission and at discharge or death, standardized clinical and laboratory data are collected.

For this study we examined all the admission data collected from January 1^st ^1990 to December 31^st ^2008. Where specific data were not collected during the entire period, we examined these data from the time such information was available, and used the admissions during this period as the denominator. Trend analysis, was done on data that were available for at least 5 consecutive years.

#### Maternity delivery data

In contrast to the paediatric ward, the hospital maternity ward record keeping was not computerized. All deliveries are recorded manually in the maternity inpatient record book supplied by the Kenyan Ministry of Health, from which we extracted the data for the study period.

#### Study population

We utilised data collected from all neonates (age ≤ 28 days at admission) [23] and non-neonatal paediatric admissions aged ≤ 5 years admitted to the hospital during the study period. Sick neonates admitted to the hospital after delivery at home were considered as out-born. Those delivered either in the district hospital or any other health facility and referred to the ward in case of ill-health were considered to be hospital births or in-born. Data extracted included:-*i) *Age and place of birth. ii) *Clinical Presentation and examination findings*: complaints: fever, cough, convulsion, diarrhoea, vomiting, jaundice, hypoxemia, respiratory distress, impaired consciousness, agitation, bulging fontanel and impaired perfusion. iii) *Clinical diagnostic syndrome*: admission discharge diagnoses recorded as: birth asphyxia, prematurity, neonatal sepsis, meningitis, neonatal jaundice and neonatal tetanus. iv) *Laboratory investigations*: culture results, full blood count, blood glucose. v) *Outcome at discharge*: dead or alive.

#### Formulation of clinical diagnoses

Routinely, all paediatric admissions to our centre are reviewed at admission, and then at least daily till discharge or death by clinicians under close supervision of a consultant paediatrician. Diagnoses of neonatal sepsis, neonatal encephalopathy, prematurity, meningitis, neonatal tetanus and neonatal jaundice were made after review of admission history, inpatient management notes and laboratory investigations at point of discharge. These diagnoses follow recognized guidelines for management of common illness with limited resources [24]. A diagnosis of invasive bacterial disease (IBD) was made on isolation of a pathogenic organism from sterile sites (blood or cerebrospinal fluid). Blood cultures were done on all neonates admitted from 1998. We therefore looked at trend in positive cultures from that date onward. Sepsis was also considered as the possible diagnosis in any newborn presenting with abnormal temperature (< 36.0°C or >37.5°C), multiple skin pustules, redness or pus discharge from the umbilicus, respiratory distress, convulsions and feeding problems. A diagnosis of neonatal jaundice was made when total serum bilirubin levels (as measured by the Neobil, Schuco International, Lyndhurst avenue London) were elevated above the threshold requiring phototherapy for the age, gestation and presenting clinical signs of the newborn [25]. Neonatal tetanus was considered in any newborn presenting with trismus or spasms occurring on stimulation or spontaneously, with or without feeding difficulties. Prematurity was considered in any neonate born before 37 completed weeks of gestation if the last monthly period was known. However where this was not known, the gestation at delivery was estimated by the admitting clinician using a simplified criteria that took into account; the head circumference, mid-upper arm circumference, breast size, ear form, gentalia, and skin texture [26]. Due to limitations of monitoring of labour in most deliveries at home and even in hospitals in resource poor settings, diagnosis of neonatal encephalopathy largely relies on history and clinical examination. Therefore, a diagnosis of neonatal encephalopathy was considered in any newborn where a difficult delivery was reported with accompanying history and signs of a poor cry, convulsions, coma, irritability and abnormal muscle tone.

#### Statistical considerations

Data were entered at admission and discharge using a FileMaker Pro database (5.5v1 Developer, FileMaker Inc, USA). We used Stata 9.2 (StatsCorp, Tx, USA) for the final analysis. Total cases of neonatal admissions and neonatal admissions by final clinical diagnosis were assembled chronologically by admission year and total non-neonatal admissions similarly assessed. They were initially examined using time series regression analysis models. A continuous variable indicating time in years from the start of observations was included in the models. The coefficient of time in the models estimates the trend in the series (the year-to-year change). A P-value <0.05 was considered significant. Likewise, we evaluated the trend in the proportions of neonatal admissions to total paediatric admissions and also examined the trend in annual maternity deliveries at the hospital. Initially we analyzed the overall neonatal admission trend for the entire 19-year period. However, from the year 2000, information about the place of delivery of all newborn admissions was systematically collected. Therefore we further analyzed the two periods (1990 to1999) and (2000 to 2008) separately.

Several assumptions are used in ordinary least square analysis [27]. These assumptions are almost always violated by longitudinal data [28,29]. We therefore used a Durbin-Watson statistic to test for serial autocorrelation that showed there were minimal (non-significant) serial autocorrelations in the raw neonatal and non-neonatal datasets (Durbin's alternative test for autocorrelation P = 0.29 & 0.15, respectively). To further correct for any autocorrelation and possible heteroscedasticity, the *Prais-Winsto*n command specifying the *Cochran-Orcutt ssesearch *option was used in the regression models. Differences in proportions were examined using a χ ^2 ^test.

In order to delineate factors associated with inpatient neonatal death, likelihood ratios (LR) were used. LR are less likely to change with prevalence of a disorder than sensitivity and specificity and may be used to combine results from multiple tests [30]. For this purpose, data from 2000 to 2008 were used because all the variables of interest were available from that period. Initially we examined the crude positive (PLR) and negative (NLR) likelihood ratios for neonatal death of each clinical indicator. Indicators with crude likelihood ratios ≥ 2.0 or ≤ 0.5 were considered potentially independent. These were then adjusted for the confounding effects of other variables in multivariate analyses using the method of Speigelhalter and Knill-Jones [31]. Thirdly practical prediction rules for inpatient neonatal deaths using indicators with adjusted likelihood ratios of ≥ 2.0 or ≤ 0.5 in multivariate analysis were constructed. Finally, we evaluated the ability of the final rules to predict inpatient neonatal death with a receiver operating curve (ROC). We have previously described this approach [32,33].

## Results

**Figure 1:**
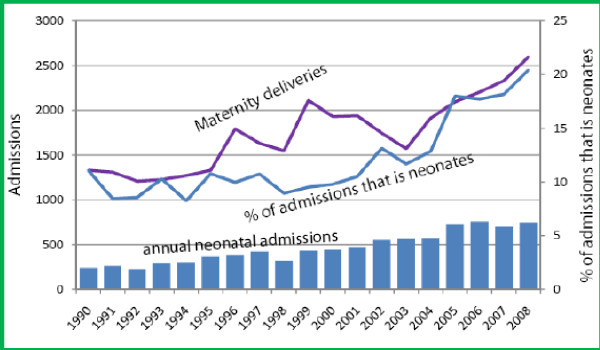
**Showing trend in:- annual neonatal admissions, neonatal admissions as a proportion of total paediatric admissions aged ≤5 years, & maternity deliveries in the hospital**.

There was a marked increase in the burden of neonatal admissions (fig1), with the total annual number of neonatal admissions significantly increasing by 211% from 240 cases in 1990 to 759 cases in 2008 (trend 34.68 (95% CI 25.60-37.76, t-stats 11.05, p < 0.001)). The proportion of neonatal admissions to total paediatrics admissions aged ≤ 5years increased from 11% in 1990 to 20% by 2008. This was significant (trend 0.83 (95% CI 0.45- 1.2, t-stats 4.63, p < 0.001) and remained significant even after correcting for annual population growth at 3% per annum (trend 0.08 (95% CI 0.02-0.14, t-stats 2.79, p = 0.01). The median age of the neonates at admission decreased significantly from 7 days (IQR 11, 3; 14 days) in 1990 to 3 days (IQR 10, 0; 10 days) by 2008 (trend -0.212 (95% Confidence interval (CI) -0.267-0.156), t-stats 8.01, p < 0.001)). Overall very young neonates (< 7days old) comprised 5400 (62%) of the total neonatal admissions. Neonates aged <7days increased from just 119 cases in 1990 to 577 cases by 2008, an increment of 385%, whilst the remainder of neonatal admissions registered an increment of only 40% over the same period.

The clinical diagnoses of neonatal sepsis, prematurity, neonatal jaundice, neonatal encephalopathy, tetanus and neonatal meningitis comprised over 75% of the inpatient neonatal admissions. Over the first 10 years (1990-1999), the number of admissions with neonatal sepsis, neonatal encephalopathy and neonatal jaundice increased significantly. However the proportion of total under five admissions that were neonatal remained unchanged (tstats 1.92, p = 0.10). In the second epoch (2000-2008), the number of neonates with diagnoses of neonatal jaundice and neonatal tetanus showed a slight decrease, while those with diagnoses of neonatal sepsis, prematurity and neonatal encephalopathy increased significantly (table 1). Although clinical diagnosis of neonatal sepsis increased the most from 1990 to 1999, increasing by 260%, from 2000, neonatal encephalopathy had the greatest increase (350%). There was no significant change in the number of neonates with invasive bacterial disease (table 1).

**Table 1:** Overall trend in burden of individual clinical diagnoses 1990-2008

Admissions annual cases							
Year	Neonatal sepsis	Culture positive	Prematurity	Neonatal encephalopathy	Neonatal jaundice	Neonatal tetanus	Neonatal meningitis
1990	49	-	50	-	-	20	7
1991	78	-	32	4	-	41	9
1992	69	-	40	5	-	26	6
1993	81	-	73	5	-	28	7
1994	100	-	59	9	-	13	4
1995	148	-	58	13	-	22	4
1996	149	-	39	11	-	32	4
1997	172	-	47	6	9	10	2
1998	134	54	41	11	35	26	1
1999	178	70	57	22	38	25	3
Trend:1990- 1999(95%CI)	13.8 (8.2-19.4)	**	0.6 (-4.1-5.3)	1.6 (0.3-2.9)	**	-1.3 (-3.0-0.5)	-0.8 (-1.0 to -0.7)
t-stats	5.78	-	0.30	3.0	-	-1.75	-12.9
Pvalue	**0.001**	-	0.77	**0.02**	-	0.12	**< 0.001**
2000	144	55	61	29	46	25	9
2001	149	52	72	37	91	27	5
2002	227	51	79	37	65	23	12
2003	234	51	79	68	88	6	14
2004	245	57	64	57	91	11	11
2005	274	75	90	100	118	12	17
2006	238	58	106	120	91	12	6
2007	276	57	97	111	84	18	9
2008	307	46	136	133	55	13	17
Trend:2000- 2008(95%CI)	16.5 (6.2-26.7)	0.0 (-4.5 -4.5)	7.3 (2.8-11.8)	14.6 (11.3-17.8)	-2.7 (-11.7-6.2)	-1.0 (-4.5-2.6)	0.5 (-0.8 -1.9)
t-stats	3.94	0.0	4.00	10.86	-0.74	-1.66	0.93
Pvalue	**0.008**	1.0	**0.007**	**< 0.001**	0.49	0.54	0.39
Overall Trend: 1990-2008 (95%CI)	13.1 (10.7-15.6)	-0.6 (-3.1 -1.9)	4.8 (2.4-7.2)	11.0 (5.7-16.3)	5.9 (1.7-10.2)	-1.0 (-1.6 to -0.4)	0.6 (0.06 -1.2)
t-stats	11.3	-0.58	4.2	4.4	2.9	-3.6	2.35
P-Value	**< 0.001**	0.58	**0.001**	**< 0.001**	**0.01**	**0.002**	**0.03**

** Trend analysis not done where data available for <5years.

The number of admissions among in-born neonates increased significantly from 40(9%) of all neonatal admissions in 2000 to 350 (47%) in 2008 (trend 32.67(95% CI 25.29-40.05, t-stats 10.83, p < 0.001), an absolute increment of 775%. In contrast, the burden of out-born ill neonates remained largely unchanged over the same period. The proportions of neonatal encephalopathy among inborn (506/1,787(28%)) was significantly higher than that among out-born (186/3732(5%), χ^2^= 600, P < 0.001).

Over the 19-year period, annual deliveries at the district hospital increased from 1,329 in 1990 to 2,597 by 2008, an increment of 95% (fig 1). The year-to-year increment was significant (trend 72.5, t-stats 5.6, p < 0.001). The projected population of women within the reproductive age group in the catchment area increased by 17% over the same period [19].

Overall, neonatal inpatient case fatality was significantly higher; 2,053/8,756 (23.5%) compared to 3,664/63,096 (5.8%) in the non-neonatal paediatric admissions aged ≤ 5years (χ^2^= 2.5e+03, P < 0.001) over the 19-year period. Of the neonatal deaths, 54 (2.6%) occurred among newborn who though admitted as neonates, died after 28 days of life. The case fatalities for the main neonatal diagnoses were; tetanus 256/390 (65.6%), prematurity 554/1,280(43.3%), neonatal encephalopathy 253/778(35.1%), meningitis 39/147 (26.5%), sepsis 547/3,252 (16.8%) and neonatal jaundice 116/811(14.3%) respectively.

**Figure 2:**
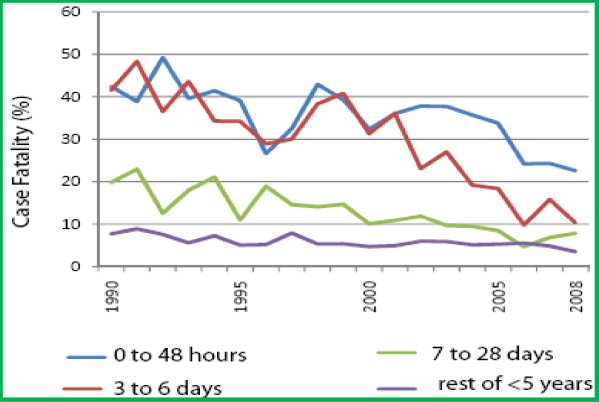
**Displaying trend in inpatient case fatality for neonates aged ≤ 48 hrs, 3 to 6 days and 7 to 28 days at admission and the rest of the paediatric admissions ≤ 5 years**.

Neonatal inpatient case fatality decreased from 30.8% in 1990 to 16.5% in 2008 with that in the rest of the ≤ 5 years decreasing from 8.0% to 3.5% respectively (fig 2). The declining trend in both were significant (tstats -5.3, p < 0.001) and (tstats -3.7, p = 0.002) respectively. However whilst annual deaths in the non-neonatal pediatric admissions aged ≤5years decreased by 31%, annual neonatal deaths increased by 67%. Overall neonatal deaths as a proportion of all ≤ 5years inpatient death increased significantly from 33% in 1990 to 55% in 2008 (tstats 6.3, p < 0.001).

From 1990 to1999 the clinical diagnosis of neonatal sepsis had the greatest increase as a cause of inpatient neonatal death (table 2). However death from neonatal encephalopathy also increased significantly over the same period. From 2000 to 2008, whilst inpatient death from the clinical diagnoses of neonatal sepsis, neonatal tetanus and meningitis declined, that from neonatal encephalopathy continued to increase significantly (table 2). The case fatality rate of the most important causes of death declined from 1990 to 2008 as follows: prematurity 50% to 30.8%, neonatal meningitis 42.9% to 11.8%, neonatal sepsis 30.6% to 5.5% and neonatal jaundice 25.7% to 3.6%. The case fatality of neonates with invasive bacterial disease also declined from 42.5% in 1998 to 23.9% in 2008. The trend of declining case fatality was significant for the clinical diagnoses of neonatal sepsis and neonatal jaundice; (tstats -9.9, p < 0.001) and (tstats -5.5, p < 0.001) respectively and among those with invasive bacterial disease, (tstats -6.5, p < 0.001). The case fatality from neonatal tetanus and neonatal encephalopathy did not change significantly over the 19 years.

**Table 2:** Overall trend in inpatient death: 1990-2008

Death annual cases										
*Year*	*All* *neonates*	*Other* *≤ 5years* *admissions*	*Neonatal* *sepsis*	*prematurity*	*Neonatal* *encephalopathy*	*Neonatal* *jaundice*	*Neonatal* *tetanus*	*Neonatal* *meningitis*	*Neonates* *aged* *<7days*	*Neonates* *aged 7-28* *days*
1990	74	149	15	25	-	-	12	3	50	24
1991	84	253	21	10	1	-	34	4	50	34
1992	67	186	25	17	1	-	15	2	54	13
1993	91	142	23	34	3	-	17	3	69	22
1994	93	245	32	30	0	-	12	2	65	28
1995	95	154	31	31	4	-	15	0	78	17
1996	90	177	35	15	4	-	22	0	60	30
1997	104	279	51	20	1	-	5	2	79	25
1998	96	174	34	17	3	9	17	1	78	18
1999	128	220	42	22	12	11	20	1	102	26
Trend 1990-99 (95%CI)	4.10 (2.73-5.48)	1.41 (-7.02-9.84)	2.95 (1.78-4.11)	0.13 (-3.05-3.31)	0.66 (0.08-1.24)	**	-1.02 (-2.36-0.33)	-0.32 (-0.64-0.01)	4.46 (2.91-6.00)	-0.22 (-1.47-1.02)
t-stats	7.04	0.40	5.99	0.10	2.68	-	-1.78	-2.29	6.82	0.43
P-Value	**0.001**	0.70	**0.001**	0.92	**0.03**	-	0.12	**0.06**	**< 0.001**	0.68
2000	104	192	26	28	10	7	17	3	87	17
2001	122	197	19	34	12	27	19	0	102	20
2002	131	218	33	42	15	13	12	3	105	26
2003	139	252	38	35	29	14	4	4	118	21
2004	128	199	33	38	19	11	6	2	108	20
2005	151	174	30	50	31	9	7	6	127	24
2006	111	197	20	35	34	5	4	1	98	13
2007	120	153	22	29	32	8	9	0	105	15
2008	124	103	17	42	42	2	9	2	111	13
Trend 2000-08 (95%CI)	-1.30 (-6.22- 3.63)	-21.56 (-41.50 -1.62)	-2.37 (-7.03-2.30)	0.07 (-2.32-2.46)	3.74 (2.93-4.54)	-2.27 (-2.69 -1.85)	-0.25 (-3.43-2.94)	-0.08 (-0.88-0.62)	4.22 (1.91- 6.53)	-5.37 (-9.40 -1.35)
t-stats	-0.64	-2.65	-.1.24	0.07	11.35	-13.14	-0.19	-0.27	4.47	-3.27
Pvalue	0.54	**0.04**	0.26	0.94	**< 0.001**	**< 0.001**	0.86	0.80	**0.004**	**0.02**
Trend 1990-2008 (95%CI)	3.31 (2.03-4.60)	-2.10 (-5.55 -1.35)	-0.77 (-2.230.69)	1.42 (0.43-2.42)	2.58 (1.79-3.37)	0.49 (-0.55-1.53)	-0.92 (-1.37 to 0.46)	0.01 (-0.17-0.18)	3.84 (2.79-4.89)	-0.51 (-0.87 to 0.15)
t-stats	5.46	-1.29	-.1.12	3.04	6.93	1.0	-4.24	0.06	7.76	-2.97
PValue	**0.001**	0.22	**< 0.001**	**0.001**	**< 0.001**	**0.01**	**0.002**	**0.03**	**< 0.001**	**0.009**

** Trend analysis not done where data available for <5years.

Most death's occurred during the first week of life, with 70% of all deaths occurring within the first forty-eight hours of life. Overall death among the very young neonates (< 7days old) was significantly higher; 1,647/5,400 (30.5%) compared to the rest of the neonatal period 406/3,356 (12.1%) (χ^2^= 252, P < 0.001) over the 19-year period. From the year 2000, neonates that died within forty-eight hours of life were more likely to be cases of neonatal encephalopathy (χ^2 ^= 11.1, P = 0.001). They were also more likely to be in-born rather than out-born (χ^2 ^= 4.5, P = 0.03). However the overall proportions of deaths among inborn cases of neonatal encephalopathy (154/506(30.4%), did not significantly differ from that among out-born cases of neonatal encephalopathy (70/186(37.6%), χ^2^= 3.22, P = 0.07). Likewise the overall case fatality rates among in-born (19.5%) and out-born (20.9%) did not differ significantly (χ^2^= 0.34, P = 0.56).

**Figure 3:**
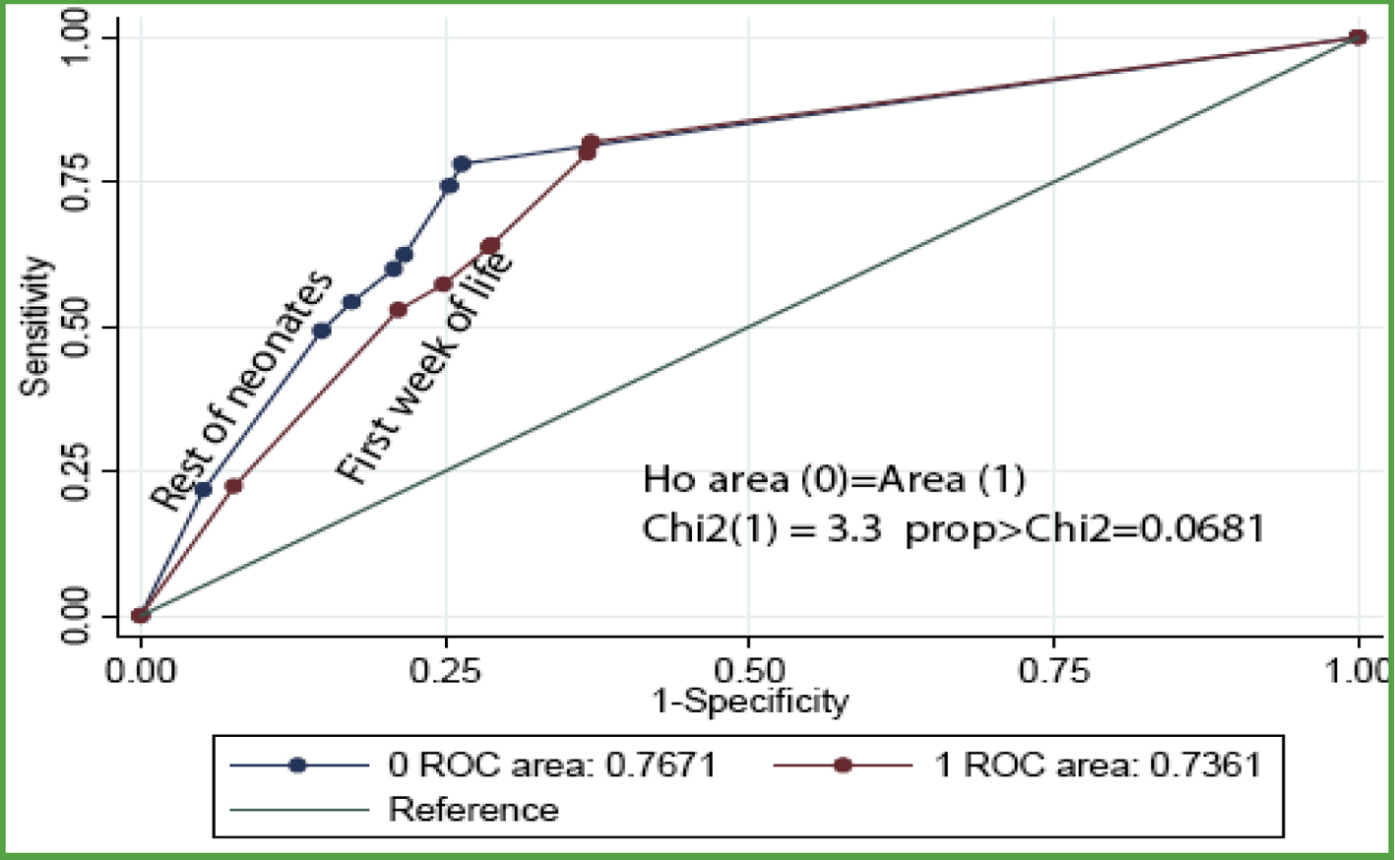
**Performance of indicators of inpatient neonatal death**.

Variables that appeared predictive of inpatient neonatal death in univariable (LR≥ 2.0) analysis were abnormal axillary temperature (< 36°C or ≥ 39.5°C), irregular breathing, respiratory rate of <30 per minute, cyanosis, oxygen saturation of <90%, temperature gradient, a weak pulse, delayed capillary refill ≥ 3 seconds, heart rate of <100 per minute, pallor, inability to breastfeed, no cry, neck stiffness, weight < 1.5 kg and blood glucose concentration >7.0 mmols/l (table 3). Of the clinical diagnoses, only neonatal tetanus and prematurity appeared predictive. However only irregular respiration, oxygen saturation of <90%, pallor, neck stiffness, weight < 1.5 kg, abnormally elevated blood glucose > 7 mmol/l, and clinical diagnosis of neonatal tetanus were independent predictors of inpatient neonatal death in multivariable analysis (table 4). There was no significant variation in the prevalence of these signs during the study period. The overall area under the ROC curve was 0.76 (95%CI 0.74-0.77), and it did not differ during and after the first week of life (χ^2^= 3.3, P = 0.07) (fig 3). Exclusion of the diagnosis of neonatal tetanus had minimal effect on the over all performance of the indicators; ROC 0.74(95%CI 0.74-0.77. the remaining six indicators predicted inpatient neonatal death with a sensitivity of 81% and a specificity of 68%.

**Table 3:** Crude predictors of inpatient neonatal death in univariable analysis

Signs	Alive	Died	Crude LR (univariable)	95%CI
Fever	1,793	250	0.54	0.48-0.61
No Fever	2,595	881	1.32	1.27 -1.37
Axilary temperature <36°C	816	533	**2.62**	2.40-2.86
36°C to 37.4°C	2,369	314	0.53	0.48- 0.59
37.5°C to 38.4°C	830	128	0.62	0.52- 0.74
38.5°C to 39.4°C	290	71	0.98	0.77- 1.26
≥39.5°C	58	40	**2.77**	1.86-4.12
Cough	749	66	1.14	1.11-1.16
No Cough	3,639	1,065	0.34	0.27-0.44
Indrawing	1,337	519	1.51	1.39-1.63
No indrawing	3,051	612	0.78	0.74-0.82
Wheeze	30	8	1.04	0.48-2.26
No wheeze	4,088	1,049	1.00	0.99-1.01
Stridor	21	6	1.10	0.44-2.72
No stridor	4,308	1,120	1.00	1.00- 1.004
Irregular breathing	280	252	**3.60**	3.08- 4.20
Regular breathing	3,545	705	0.80	0.77- 0.83
No Deep breathing	3,516	858	0.903	0.88-0.93
Difficulty in breathing	1,240	443	1.37	1.27-1.49
No difficulty in breathing	2,098	426	0.78	0.73-0.84
Nasal flaring	589	238	1.43	1.25-1.63
No nasal flaring	2,661	682	0.91	0.87- 0.94
BCG scar	885	103	0.43	0.36- 0.52
No BCG scar	2,814	898	1.18	1.15- 1.21
Respiratory rate (per minute) <30	212	181	**3.33**	2.76-4.01
30 to 59	2,894	591	0.80	0.75-0.84
60 to 80	969	264	1.06	0.94 -1.20
> 80	236	70	1.16	0.89-1.50
Cyanosis	205	208	**3.90**	3.25- 4.68
No Cyanosis	4,123	918	0.86	0.83-0.88
Oxygen saturation (%) <90	605	457	**2.94**	2.66- 3.26
90 to 100	3,072	666	0.69	0.66-0.72
Diarrhoea	66	11	0.65	0.34 -1.22
No diarrhoea	4,322	1,120	1.01	1.00-1.01
Vomiting	230	26	0.44	0.29-0.65
No Vomiting	4,158	1,105	1.03	1.02-1.043
Decreased skin turgor	135	46	1.21	0.87-1.68
Normal skin turgor	3,012	839	0.99	0.97- 1.01
Sunken eyes	59	17	1.13	0.66-1.93
No sunken eyes	3,851	978	1.00	0.99-1.08
Temperature gradient	443	297	**2.48**	2.18-2.82
No temperature gradient	3,087	657	0.79	0.75-0.82
Weak pulse	197	226	**4.43**	3.72-5.28
Normal pulse	3,004	606	0.78	0.74-0.81
Capillary refill time(seconds) <1	2,578	449	0.67	0.62-0.72
1 to 3	1,670	566	1.31	1.22-1.40
> 3	80	109	**5.25**	3.96-6.95
Heart rate(per minute) <100	130	124	**3.566**	2.82- 4.52
100 to 180	3,077	725	0.881	0.85-0.92
> 180	558	158	1.059	0.90-1.5
Pallor	225	170	**2.93**	2.43-3.54
No Pallor	4,163	961	0.90	0.87-0.92
breastfeeding	2,883	369	0.49	0.45-0.54
Not able to breastfeed	1,442	758	**2.02**	1.90 -2.14
cry	3,184	614	0.77	0.73-0.81
No cry	642	344	**2.14**	1.92 -2.39
Bulging fontanel	45	19	1.77	0.99- 2.87
Normal fontanel	3,767	935	0.99	0.98- 1.00
Neck stiffness	29	26	**3.48**	2.06- 5.88
No neck stiffness	4,286	1,087	0.98	0.97-0.99
irritable	109	55	1.97	1.43 2.70
Not irritable	4,007	1,001	0.97	0.96 -0.99
Convulsions	247	45	0.68	0.50- 0.93
No convulsions	3,513	964	1.02	1.01-1.04
Weight(kg) <1.5	367	381	**4.18**	3.68-4.74
1.5 to <2.0	492	150	1.23	1.03-1.45
2.0 to <2.5	769	166	0.87	0.74-1.01
≥2.5	2,727	386	0.57	0.52-0.62
Age in days 0 to 2	1,335	619	1.73	1.62-1.85
3 to 6	808	231	1.07	0.94- 1.22
7 to 28	1,615	156	0.36	0.31- 0.42
Neonatal encephalopathy	298	150	1.81	1.51-2.17
Not neonatal encephalopathy	2,888	737	0.92	0.89-0.95
Neonatal tetanus	47	69	**5.27**	3.67-7.58
Not neonatal tetanus	3,139	818	0.94	0.92- 0.96
preterm	289	262	**3.26**	2.80-3.78
Not preterm	2,897	625	0.78	0.74-0.81
Neonatal sepsis	1,332	199	0.54	0.47- 0.61
Not neonatal sepsis	1,854	688	1.33	1.27- 1.40
meningitis	55	19	1.24	0.74- 2.08
Not meningitis	3,131	868	1.00	0.99- 1.01
Jaundice	509	86	0.61	0.49-0.75
Not jaundice	2,667	809	1.08	1.05-1.10
Blood glucose level(mmols/l) < 2.6	705	279	1.44	1.28-1.62
≥ 2.6 to ≤ 7.0	2,327	422	0.66	0.61-0.71
> 7.0	236	199	**3.06**	2.57-3.64
Culture (blood or CSF) Positive	370	171	1.81	1.527 to 2.133
Negative	3,437	804	0.91	0.886 to 0.942

**Table 4:** Predictors of inpatient neonatal death (Multivariable analysis of crude predictors with LR ≥ 2.0)

Signs	Alive	Died	Crude LR (univariable)	95%CI	Adjusted LR (multivariable)
Irregular breathing	280	252	**3.60**	3.08- 4.20	**1.93**
Oxygen saturation (%) < 90	605	457	**2.94**	2.66- 3.26	**2.00**
Pallor	225	170	**2.93**	2.43-3.54	**2.49**
Neck stiffness	29	26	**3.48**	2.06- 5.88	**3.42**
Weight(kg) < 1.5	367	381	**4.18**	3.68-4.74	**4.03**
Blood glucose level(mmols/l) > 7.0	236	199	**3.06**	2.57-3.64	**2.14**
Neonatal tetanus	47	69	**5.27**	3.67-7.58	**7.38**

## Discussion

District health facilities play a pivotal role in the health care delivery system in resource poor countries, acting both as primary referral centres and also coordinating care at the peripheral health facilities [34,35]. The nature and composition of inpatient burden at this level may reflect the community burden, more so than that at the larger referral hospitals (provincial, national, and teaching hospitals) that are usually situated in urban centres and thus far removed from the rural communities. However no study has analysed secular trends of neonatal admissions and outcome in rural district hospitals from resource poor countries in general and Sub-Saharan Africa in particular. Our data shows that neonatal admissions both in absolute numbers and as a proportion of total paediatric admissions have substantially increased over the last 19 years. The age and pattern of neonatal admissions appears to have also changed, with much younger neonates and an increase in the cases of neonatal encephalopathy.

We found that although the total annual paediatric admissions increased markedly over the first 10 years of our surveillance (1990 to 1999), the proportions of neonatal admissions remained unchanged. Over the following nine year period (2000 to 2008) while neonatal admissions continued to increase markedly, non-neonatal admissions remained stable and even declined. While the increase in neonatal sepsis (almost three fold) accounted for much of the increase during the first 10 years of this surveillance, from 2000 onward neonatal encephalopathy was the largest increment.

It is worth noting that while hospital deliveries nearly doubled, the projected population of women within the reproductive age group increased marginally over the 19 years [19]. This may thus signify increased utilisation. The results may further support a previous research finding that pregnant women, even in rural settings would prefer skilled attendance during delivery [36]. Increasing coverage of skilled deliveries should be one of the core strategies for reducing neonatal mortality in developing countries [3]. However given the finding of higher cases of neonatal encephalopathy among hospital deliveries, studies to examine barriers of timely uptake of such facilities and how they can be eliminated are needed.

Encouragingly, this study shows a significant drop in inpatient case fatalities in both neonatal and non neonatal admissions aged ≤ 5 years. This trend seems to be mirrored at national level where under five mortality rates appear to have initially increased from 89/1000 in 1990, to 114/1000 by the year 2003, with steady reduction to 74/1000 live births by the year 2008 [37,38]. It is plausible that the initial increase was occasioned by economic deterioration in the late 1980s' through to 2002 resulting in poor quality of life and reduction in government investment in health care. The periods from 2003 onward saw renewed economic growth and increased government spending in health care [38]. Importantly our finding that there was no significant variation in the prevalence of signs indicative of severe illness or high likelihood of inpatient death over the this period, denotes that the decline in case fatality may be due to improving care rather than admission of less severely ill children. However, although inpatient neonatal and non neonatal case fatality rates decreased over the 19 years, the actual numbers of neonatal inpatient deaths markedly increased as a result of the higher admissions. Importantly, neonatal deaths as a proportion of all inpatient deaths in children aged ≤ 5 years nearly doubled. It is particularly noteworthy that neonatal encephalopathy was the most rapidly increasing cause of inpatient neonatal deaths over the entire 19-year period. Importantly while the case fatality from neonates with IBD or a clinical diagnosis of neonatal sepsis declined significantly, that from neonatal encephalopathy remained high. In the sub-Saharan African region, though neonatal encephalopathy and birth related complications are thought to be responsible for nearly a quarter of all neonatal deaths, the bulk of neonatal deaths is thought to be due to infectious causes (sepsis) [1,3,39]. This increasing prominence of neonatal encephalopathy over time with declining deaths from other major neonatal conditions especially sepsis has not been previously reported. Surprisingly death among neonates with neonatal encephalopathy (whether in-born or out-born) did not differ significantly. However the higher composition of neonatal encephalopathy among in-borns suggests that hospital deliveries may be occurring either for complicated births or where attempted home delivery has failed. It is therefore possible that cases of neonatal encephalopathy from home may represent just a minority of the mild and moderately affected newborn, with the severe ones dying soon after birth before reaching hospital care. Of note too is the high case fatality from neonatal tetanus and prematurity. Lack of ventilatory support and parenteral nutrition at our centre may partly account for this. However this finding may underscore the role of primary prevention if significant gains in achieving sustained reduction in neonatal morbidity and mortality are to be made especially where the three conditions (neonatal encephalopathy, neonatal tetanus & prematurity) are concerned.

There are several possible factors that could account for the increase in neonatal admissions and hospital deliveries with stabilization or even decline in non-neonatal admissions. Firstly is the sustained high fertility rate (estimated to be above 6.0) in the catchment area [40]. Secondly, a major cause of non-neonatal admissions notably malaria, has declined [11]. This could be due to a combination of factors such as increased coverage of insecticide treated mosquito nets and change to a more effective anti-malarial drug [13]. Moreover from 2001 introduction of *Haemophilus influenzae *type b conjugate vaccine led to a noticeable drop in cases admitted with IBD [9].

Trends in health facility utilization over the last two decades in Kenya need to be put into context considering that from late 1980 s, the country introduced user fees in public health facilities [41,42]. This was due to a combination of factors including poor economic performance, inadequate financial resources, declining budget allocations and international donor pressure [41]. Facilities set user fees locally with the support of health facility committees. Revenue collected was returned to the district level and facilities developed detailed plans for spending 75% of the revenue. A waiving policy to protect the poor was put in place, and children below five years were exempted from most charges, but in reality waiving and exemption mechanisms hardly existed [41]. Although there have been attempts towards lowering the user fees at lower level facilities (community dispensaries and health centres), this has not been effected at district hospital level. Evidence on user fees and other out of pocket payments in Kenya suggest that health care charges are a significant barrier to access [42,43], and that they can push households into poverty [43]. Indirect costs such as transport and potential loss of income are also important determinants of care seeking. It is therefore highly plausible that the trend we describe here has been greatly attenuated by combination of user fees, other out-of pocket payments and non financial barriers to accessing healthcare.

In order to reduce paediatric mortality in regions with limited resources, there have been attempts to develop diagnostic and treatment algorithms that target the principal causes of death in children [16,44,45]. The WHO developed a thirteen indicators sick child chart aimed at identifying severely ill children in need of intensive treatment or urgent referral [16]. This largely identified children with acute respiratory infections, malaria, measles, diarrhoeal diseases and malnutrition. Although neonatal deaths constitute close to 40% of all inpatient paediatric deaths in such regions, neonatal illness did not feature prominently in the development of the said chart. Our study demonstrates that simple clinical variables of irregular breathing, oxygen saturation <90%, pallor, weight <1.5 kg, blood glucose > 7.0 mmol/l and neck stiffness can predict inpatient neonatal death with good sensitivity and reasonable specificity. These variables may potentially aid in neonatal triage and thus help to reduce inpatient neonatal death. A recent multicentre study looking at clinical signs that predict severe illness in neonates and young infants ≤ 2 months old found that the signs of history of difficulty in feeding, history of convulsion, movement only when stimulated, respiratory rate of sixty breaths or more per minute, severe chest wall in drawing and abnormal temperature (≥ 37.5°C or < 35.5°C) predicted severe illness with a sensitivity of 85% and specificity of 75% [44]. However we did not find these variables to be independent predictors of inpatient neonatal death. This may suggest that signs that predict severe illness may differ from those that predict inpatient neonatal death. More studies are needed to validate the signs that we found to be predictive.

A major limitation of this study is that before 2000, information on place of delivery was not routinely collected making it difficult to compare long term trends between home deliveries and the hospital births. However given that at the start of 2000 only 40/442 (9%) of all neonatal admission were in-born, and the proportion increased thereafter, it is likely that in the preceding years hospital births were unlikely to be much higher than the 2000 figure. We were also unable to estimate the minimum incidence of neonatal admissions due to lack of information on live births in the catchment area. However we controlled for this by looking at trend in neonatal admissions as a proportion of total under five paediatrics admissions, and further adjusting for annual population growth rate.

## Conclusion

In conclusion, this study provides clear evidence of increase in inpatient neonatal burden in a district hospital coupled, with an increase in maternity deliveries in a resource poor region. Encouragingly, inpatient case fatality reduced significantly. However neonatal deaths now comprise close to 60% of all inpatient deaths in children aged ≤ 5 years and neonatal encephalopathy is a rapidly increasing cause of inpatient death. Our findings have several implications at facility, national and global levels. At the facility level measures should be put in place to ensure adequate equipment and trained personnel to handle emergency obstetric care, recognize and manage common neonatal conditions and perform simple resuscitation measures especially in cases of neonatal encephalopathy. Likewise audit of neonatal admissions and outcome should be encouraged at all health care facilities. At the national and international level sustained political will is required to formulate and implement deliberate policies aimed at reducing newborn morbidity and mortality and to mobilize and direct resources towards implementing and sustaining such policies. The place of district and community level health facilities in reducing neonatal morbidity and mortality in developing regions should also be emphasised and committed steps taken to ensure they are appropriately staffed and equipped. Finally, by further highlighting simple variables predictive of inpatient neonatal death, this study also provides possible areas to be targeted in tackling the high level of inpatient neonatal deaths in rural district hospitals.

## Declaration of commercial interest

None.

## Competing interests

The authors declare that they have no competing interests.

## Guarantor/sponsor

Wellcome trust.

## Role of sponsor

The sponsor played no role in study design, data collection, and analysis and manuscript preparation

## Authors' contributions

MKM designed the study, collected and analyzed the data and wrote the draft. HG participated in data analysis. ME, JB, NM & CC supervised inpatient care and assisted in collection and data analysis. JC reviewed the manuscript and provided contextual insights on affordability and payment for health in Kenya. NP, LM, PK & KM participated in overall study conception and draft writing. CN suggested the study, participated in study design, and was involved in data analysis and preparation of the final manuscript for submission. All authors critically reviewed the final manuscript.

## Pre-publication history

The pre-publication history for this paper can be accessed here:

http://www.biomedcentral.com/1471-2458/10/591/prepub
